# A new effLuc/Kate dual reporter allele for tumor imaging in mice

**DOI:** 10.1242/dmm.052130

**Published:** 2025-01-31

**Authors:** Latifa Bakiri, Mélanie Tichet, Carolina Marques, Martin K. Thomsen, Elizabeth A. Allen, Stefanie Stolzlechner, Ke Cheng, Kazuhiko Matsuoka, Massimo Squatrito, Douglas Hanahan, Erwin F. Wagner

**Affiliations:** ^1^ Laboratory Genes and Disease, Department of Laboratory Medicine, Medical University of Vienna, Vienna (MUW), Spitalgasse 23, 1090 Vienna, Austria; ^2^ Ludwig Institute for Cancer Research, Lausanne Branch; Swiss Institute for Experimental Cancer Research (ISREC), EPFL; Swiss Cancer Center Leman (SCCL); Agora Translational Cancer Research Center, Rue du Bugnon 25A, 1005 Lausanne, Switzerland; ^3^ Seve Ballesteros Foundation Brain Tumor Group, Spanish National Cancer Research Centre, Melchor Fernández Almagro, 3, 28029 Madrid, Spain; ^4^ Department of Biomedicine, Aarhus University, Høegh-Guldbergs Gade 10, 8000 Aarhus, Denmark; ^5^ Laboratory Bone Cancer Metastasis, Cellular and Molecular Tumor Biology, Center for Cancer Research, Medical University of Vienna (MUW), Spitalgasse 23, 1090 Vienna, Austria; ^6^ Laboratory Genes and Disease, Department of Dermatology, Medical University of Vienna (MUW), Spitalgasse 23, 1090 Vienna, Austria

**Keywords:** Dual reporter allele, Genetically modified mouse model, eff Luciferase, mKate, Knock-in

## Abstract

Genetically engineered mouse models (GEMMs) are instrumental for modelling local and systemic features of complex diseases, such as cancer. Non-invasive, longitudinal cell detection and monitoring in tumors, metastases and/or the micro-environment is paramount to achieve a better spatiotemporal understanding of cancer progression and to evaluate therapies in preclinical studies. Bioluminescent and fluorescent reporters marking tumor cells or their microenvironment are valuable for non-invasive cell detection and monitoring *in vivo*. Here, we report the generation of a dual reporter allele allowing simultaneous bioluminescence and fluorescence detection of cells that have undergone Cre-Lox recombination in mice. The single copy knock-in allele in the permissive collagen I locus was evaluated in the context of several cancer GEMMs, where Cre expression was achieved genetically or by ectopic virus-mediated delivery. The new reporter allele was also combined with gene-targeted alleles widely used in bone, prostate, brain and pancreas cancer research, as well as with alleles inserted into the commonly used Rosa26 and collagen I loci. This allele is, therefore, a useful addition to the portfolio of reporters to help advance preclinical research.

## Teaser

**Summary:** A single-copy effLuc/Kate dual reporter knock-in allele for non-invasive longitudinal monitoring and FACS-isolation of cells that have undergone Cre-mediated recombination is evaluated in mouse models for bone, prostate, brain and pancreas cancer.

## INTRODUCTION

Genetically engineered mouse models (GEMMs) designed to recapitulate complex aspects of cancer in a whole organism have proven useful in dissecting early-disease stages and progression, as well as assessing treatment modalities (Kersten et al., 2017). New technological developments, such as the design of conditional alleles, where gene modifications are controlled spatially and/or temporally, as well as the use of CRISPR/Cas9-based genome editing for rapid germline or somatic gene manipulations, have further accelerated preclinical cancer research (Katti et al., 2022). These developments have increased the pool of targeted alleles that can be combined to improve modelling of human pathologies and allowed high-throughput *in vivo* screens for drugs and tumor-modulating genes (Chow and Chen, 2018; Dubrot et al., 2022; Xia et al., 2022).

Imaging is a crucial tool in life science and medical research, providing detailed insights into biological structures and processes that are otherwise opaque. Advanced imaging techniques, such as intra-vital endoscopy and microscopy as well as MRI and CT scans, enable researchers to observe organs, tissues and cells in a living organism, track disease progression and evaluate the effects of treatments in real-time. Furthermore, non-invasive imaging technologies can be used to monitor tumor development and progression longitudinally and, thereby, reduce both the number of animals and their distress, concordant with two principles of animal welfare in research (Koo et al., 2006).

The inclusion of life-compatible cell-tracking methods within imaging techniques has been evolving for decades. Fluorescence or bioluminescence optical imaging is commonly used in mouse models due to its sensitivity, relatively low cost and user-friendly features. The most robust methods rely on the inclusion of alleles harboring constitutively expressed or time/tissue-switchable fluorescent protein or luciferase-encoding genes in GEMMs. A wide range of fluorescent proteins have been isolated from cnidarias, and developed and improved for color, brightness, photo-stability and performance in various imaging applications (Kawamoto et al., 2000; Novak et al., 2000; Srinivas et al., 2001). For example, mKate2 is a far-red fluorescent protein applicable for multicolor imaging as well as to image tissues with high endogenous green fluorescence, and useful to detect transplanted tumor cells in hairless skin with a low-power optical imaging system (Tanida et al., 2014; Zhou et al., 2018). Moreover, since the first report describing the usefulness of firefly luciferase to image transgene expression *in vivo* (Contag et al., 1997), bioluminescence imaging has been widely used in preclinical research to study tumor growth and metastasis (Obenauf et al., 2015; Tichet et al., 2015). By tagging cancer cells or, more recently, cells of the tumor microenvironment, bioluminescence imaging has enabled real-time tracking of disease progression and treatment response in live animal models (Lyons et al., 2003; Kim et al., 2015; Rabinovich et al., 2008; Galliverti et al., 2018; Chryplewicz et al., 2022). Luciferases, found in a wide range of organisms from bacteria to insects, are light-emitting enzymes that, along with their substrates, have been the focus of extensive bioengineering efforts (Yao et al., 2018). For example, color-shifted luciferase genetic derivatives have recently allowed simultaneous bioluminescence imaging of two co-transplanted cell populations *in vivo* (Torres Chavez et al., 2024).

In this article, we describe the generation and *in vivo* cancer research applications of an enhanced firefly luciferase/mKate dual reporter. This new reporter is integrated in the collagen I locus, can be combined with popular genetically engineered alleles used in cancer research including Rosa26-based reporters, and allows simultaneous bioluminescence imaging and fluorescence imaging of cells that have undergone Cre-Lox recombination.

## RESULTS AND DISCUSSION

### Engineering a dual bioluminescent/fluorescent knock-in reporter

A β-actin promoter-driven cassette comprising loxP-STOP-loxP (LSL), enhanced firefly luciferase (effLuc) and mKateS158A (LSL-effLuc-Kate) was cloned into a modified version of the pgk-ATG-frt shuttle plasmid (Addgene plasmid #20734) and electroporated together with a flippase eighth generation clone (FLPe) transient expression vector pCAGGS-flpE-puro (Addgene plasmid #20733) into KH2 ES cells to achieve site-specific FLPe-mediated recombination into the flippase recognition target (frt) frt-hygro-pA ‘homing’ cassette located downstream the permissive *Col1a1* locus (Beard et al., 2006). The resulting hygromycin-resistant *flp-in* ES clones carried a single-copy integration in the *Col1a1* locus of a dual effLuc-Kate reporter that can be activated *in vitro* and *in vivo* by Cre-mediated deletion of the LSL cassette (Fig. S1A). Upon establishing germline transmission, the functionality of the dual reporter was first tested using immunofluorescence and bioluminescence in primary fibroblasts that had been isolated from the ear of an adult mouse carrying the *Col1a1*^LSL-effLuc-Kate^ allele and infected *in vitro* with an Cre recombinase-carrying adenovirus (Fig. S1B).

### Assessing the LSL-effLuc-Kate reporter in bones and bone tumors

The *Col1a1*^LSL-effLuc-Kate^ allele was next combined with the Osx-Cre allele (Rodda and Mcmahon, 2006) to activate reporter expression in all cells of the osteoblastic lineage. While bioluminescence was detected in live *Col1a1*^LSL-effLuc-Kate^; Osx-Cre^+^ double transgenic mice, particularly in areas where the skeleton was close to the skin, no specific Kate-derived red fluorescence was detected in this setting (Fig. S1C,D, top panels). Bioluminescence and red fluorescence were, however, clearly detectable after sacrificing the mice and exposing the skeleton (Fig. S1C,D, bottom panels). The new *Col1a1*^LSL-effLuc-Kate^ allele is, therefore, functional *in vitro* and *in vivo*.

**Fig. 1.**
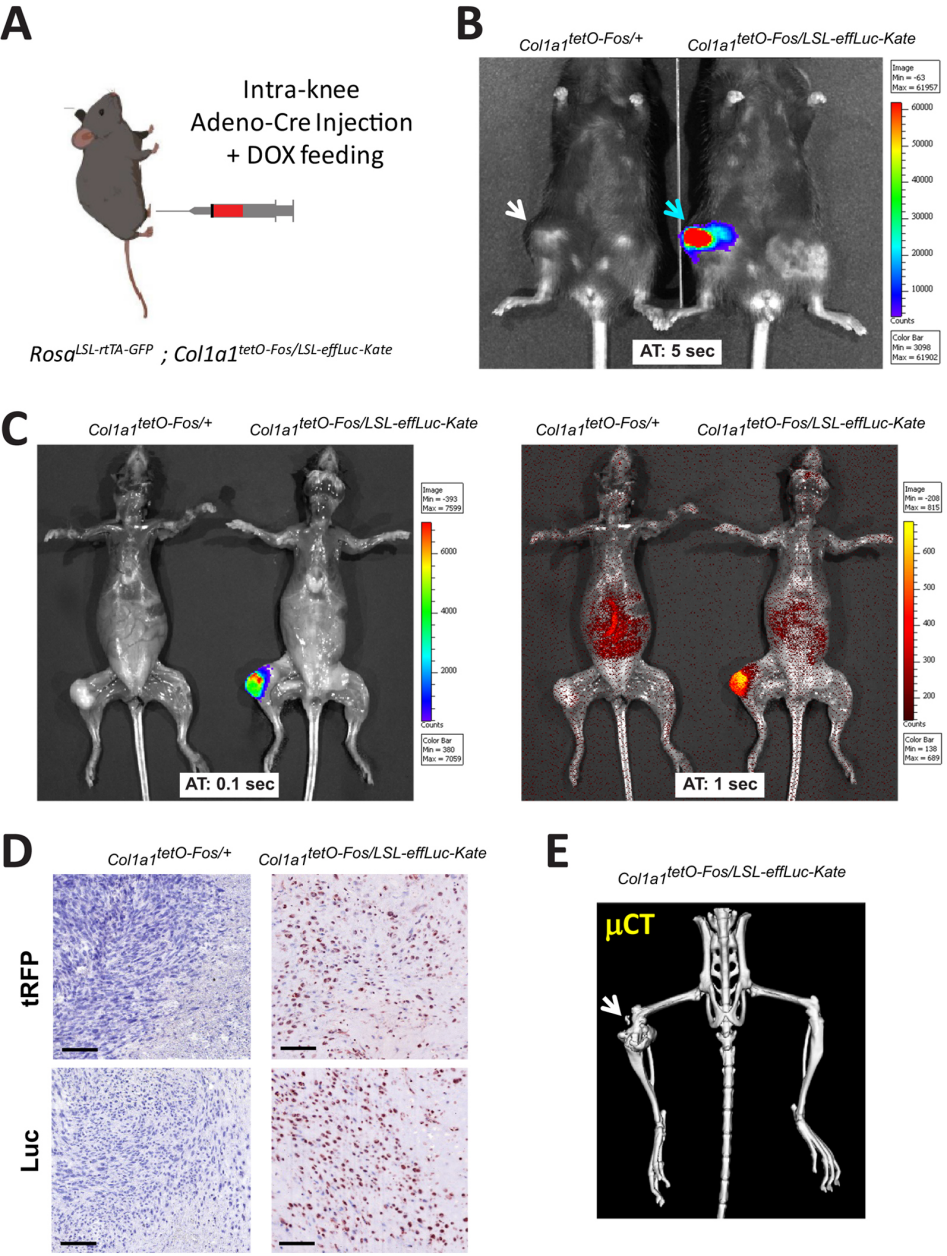
**A dual bioluminescent−fluorescent knock-in reporter allele – assessment in bone.** (A) Schematic of the experimental setup. Adeno-Cre virus (10 µl, 10^8^ pfu) was injected under the knee capsule of anesthetized 8-week-old mice carrying a Rosa^LSL-rtTA-GFP^ allele, a tetracycline (Tet)-inducible Col1a1^TetO-Fos^ allele and, eventually, the *Col1a1*^LSL-effLuc-Kate^ reporter. Mice were then fed a Dox-containing diet until sacrifice. (B) Live IVIS imaging for luciferase 4 weeks after Adeno-Cre inoculation into female mice carrying the *Col1a1*^LSL-effLuc-Kate^ dual reporter (right) compared with reporter-negative littermates (left). Arrows indicate tumors in the Adeno-Cre-injected knees. (C) IVIS imaging for luciferase (left) and red fluorescence (right) in mice as shown in A after sacrifice and skin removal. Arrows indicate IVIS-positive tumors in Adeno-Cre-injected knees. Experiments were performed at least three times using male and females. (D) Representative images showing knee tumor sections stained for turboRFP (tRFP) to detect mKate (top panels) or luciferase (Luc) (bottom panels) obtained from Adeno-Cre-injected reporter-positive (right) or negative (left) mice. Magnified areas of each image are shown in the insets. Scale bars: 100 µm. (E) Representative 3D reconstitution of hind legs following *ex vivo* micro-CT of an Adeno-Cre-injected *Col1a1^TetO-Fos/LSL-effLuc-Kate^* mouse. Arrow indicates the Cre injection/tumor site. AT, IVIS signal acquisition time.

The *Col1a1*^LSL-effLuc-Kate^ reporter was next combined with the previously published Col1a1^TetO-Fos^ allele (Briso et al., 2013) – in which a tetracycline (Tet)-inducible c-Fos cassette was inserted in the Col1a1 locus using the same strategy as the Col1a1^LSL-effLuc-Kate^ reporter – as well as the Rosa^LSL-rtTA-GFP^ allele that allows expression of a reverse tetracycline transactivator (rtTA) and, hence, enables doxycycline (Dox)-induced temporal control of c-Fos expression upon Cre-mediated excision of the LSL cassette (Belteki et al., 2005). This bi-allelic modification of the *Col1a1* locus in the same mouse had no overall effect on viability or health. Eight-week-old mice were injected under the right kneecap with an adenovirus constitutively expressing the Cre recombinase, and then fed a Dox-containing diet (Fig. 1A). Littermates carrying only the Col1a1^TetO-Fos^ and the Rosa^LSL-rtTA-GFP^ alleles were included as a control for baseline red fluorescence and bioluminescence. Four weeks after injection, palpable tumors were observed at the injection sites for both genotypes expressing Fos; however, bioluminescence was only detected in live mice additionally carrying the *Col1a1*^LSL-effLuc-Kate^ dual reporter (Fig. 1B). Moreover, red fluorescence from the mKate reporter was only detected when using the in-vivo imaging system (IVIS) in the same luciferase-positive tumors after sacrifice and following skin removal (Fig. 1C). Immunohistochemistry (IHC) confirmed that only knee tumors from mice carrying the *Col1a1*^LSL-effLuc-Kate^ dual reporter expressed mKate and luciferase (Fig. 1D), while micro-CT analyses indicated that the lesions contained calcified as well as uncalcified areas, thus, pointing towards an osteoblastic origin (Fig. 1E).

Taken together, these results indicate that the *Col1a1*^LSL-effLuc-Kate^ reporter allele is functional. When activated by Cre expression or by orthotopic Adeno-Cre delivery, the dual reporter emits luciferin bioluminescence or red fluorescence that is readily detectable *in vivo* or *ex vivo*, respectively. In addition, the new knock-in reporter allele can be combined in the same mouse with other genes integrated into the *Col1a1* locus, as well as one integrated into the *Rosa26* locus.

### Applicability in cancer models initiated by orthotopic intra-organ Cre delivery

**Fig. 2.**
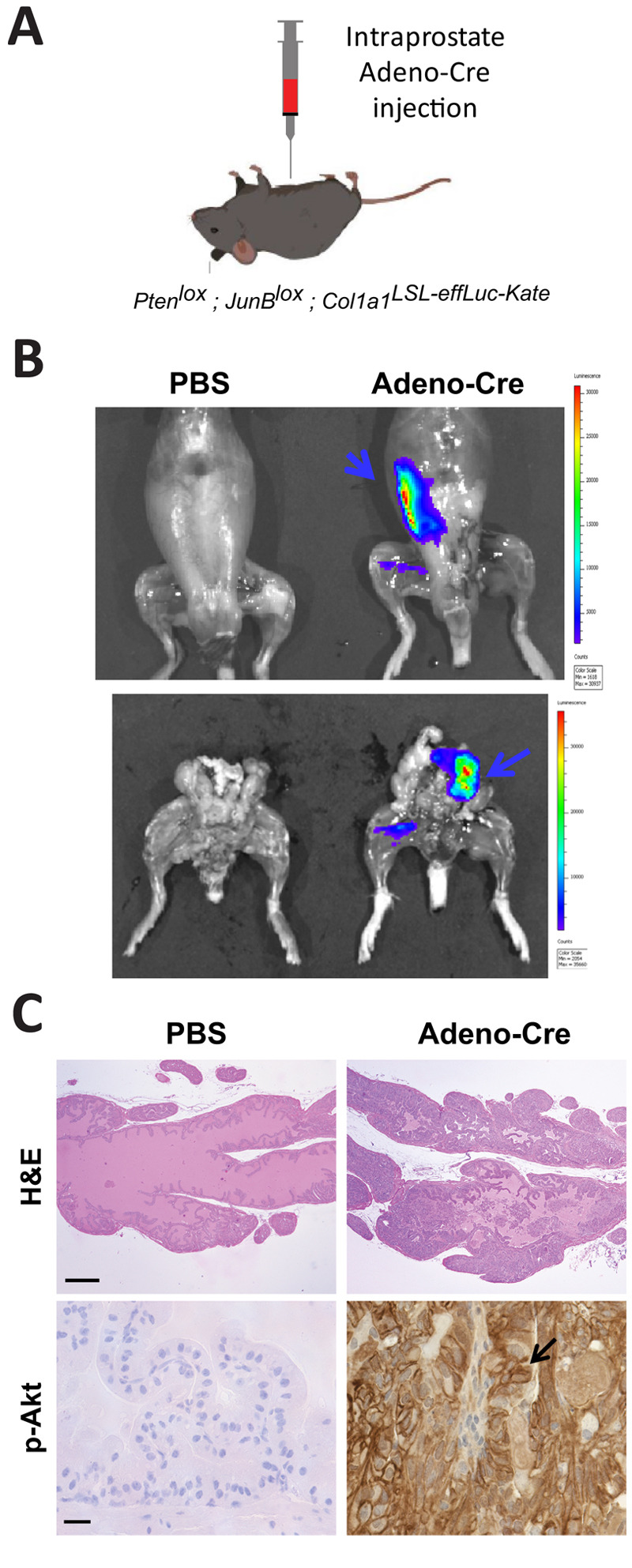
**Imaging of prostate cancer initiated by orthotopic intra-organ Adeno-Cre injection.** (A) Schematic of the experimental setup. Adeno-Cre virus (10^8^ pfu) or the equivalent volume of PBS (25 µl) (control) was injected in the anterior prostate of anesthetized 7-week-old male mice homozygote for Pten*^lox^* and Junb*^lox^* and carrying the *Col1a1*^LSL-effLuc-Kate^ dual reporter. (B) IVIS imaging 12 months after PBS or Adeno-Cre injection. Top images show the abdominal area after skin removal. Bottom images show the same mice following exposure of the male genital tract. Arrows indicate abdominal (top) and genital (bottom) areas with a specific luminescence signal. (C) H&E and immunohistochemistry staining against phosphorylated Akt (p-Akt) in dorsolateral prostate sections from PBS (control) or Adeno-Cre-injected mice as shown in A. The arrow indicates a p-Akt positive region in the neoplastic lesion. Scale bars: 100 µm (H&E), 20 µm (p-Akt).

We next evaluated the reporter in two mouse models, in which tumors had been induced by orthotopic Cre-delivery in two different internal organs, the prostate and the brain. The *Col1a1*^LSL-effLuc-Kate^ reporter was combined with Pten*^lox^* and Junb*^lox^* alleles amenable to Cre-mediated homozygous deletion of *Pten* and *Junb* (Fig. 2A). In this model, surgical inoculation of adenoviral particles carrying Cre recombinase into the anterior lobe of the prostate (Riedel et al., 2018; Thomsen et al., 2015) resulted in the development of prostate tumors in adult males (Thomsen et al., 2015). Twelve months post infection, specific bioluminescence was only detected in Cre-injected mice after sacrificing the mice and removing the skin (Fig. 2B, top panel), whereas no red fluorescence was detected by using the IVIS. Further dissection indicated that the abdominal signal originated from the genital area (Fig. 2B, bottom panel), while histological analyses confirmed the presence of phosphorylated Akt (p-AKT)-positive tumors in the dorsolateral prostate (Fig. 2C). Similar results were obtained when Cre was expressed constitutively from a transgenic allele under the control of the prostate-specific antigen promoter (Psa-Cre). Bioluminescence was weak and only detected after sacrifice and skin removal in 10-month-old Pten*^lox^*; Junb*^lox^*; Psa-Cre^+^ mice carrying the reporter (Fig. S2A). Diffuse mKate and weaker luciferase IHC staining were detected in prostate tissue sections from Pten*^lox^*; Junb*^lox^*; Psa-Cre^+^ mice carrying the *Col1a1*^LSL-effLuc-Kate^ dual reporter (Fig. S2B).

**Fig. 3.**
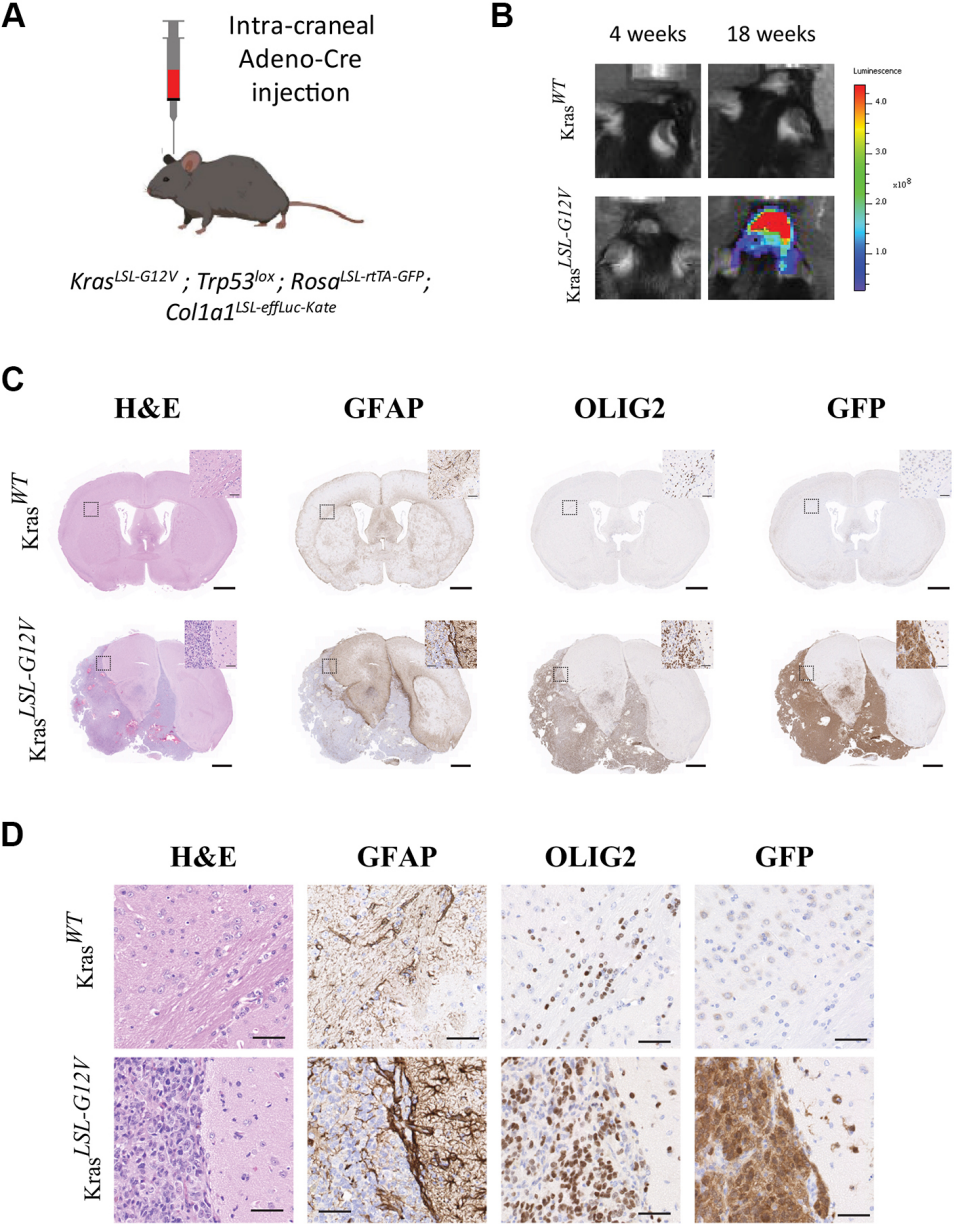
**Imaging of brain cancer initiated by orthotopic intra-organ Adeno-Cre injection.** (A) Schematic of the experimental setup. Mice homozygous for Trp53*^lox^* and Rosa^LSL-rtTA-GFP^, and carrying the *Col1a1*^LSL-effLuc-Kate^ dual reporter and a wild-type or Kras*^LSL-G12V^* allele were anesthetized and injected intracranially with 2×10^8^ pfu of Adeno-Cre virus at 40 days of age. Male Kras*^WT^* and Kras*^LSL-G12V^* mice (*n*=6) from three independent cohorts and experiments were longitudinally followed. (B) Representative IVIS images obtained 4 and 18 weeks (endpoint) after intracraneal Adeno-Cre injection of mice homozygous for Tp53*^lox^* and Rosa^LSL-rtTA-GFP^ and carrying the *Col1a1*^LSL-effLuc-Kate^ dual reporter and either wild-type (Kras*^WT^*, top images) or mutant Kras*^LSL-G12V^* (bottom images) allele. Bottom right image shows luciferase signals in the head of the mouse carrying the reporter and the Kras^LSL-G12V^ alleles. (C,D) H&E staining and immunohistochemistry (IHC) staining against GFAP, OLIG2 or GFP in sagittal brain sections from Adeno-Cre-injected mice as shown in B at 18 weeks (top panels: Kras*^WT^*, bottom panels: Kras*^LSL-G12V^*). Boxed areas are shown magnified in the top right of each image as well as separately in D. Scale bars: 1 mm, 50 µm (inserts).

The *Col1a1*^LSL-effLuc-Kate^ reporter was next combined with the Rosa^LSL-rtTA-GFP^, Kras*^LSL-G12V^* and Trp53*^lox^* alleles to allow simultaneous Cre-mediated induction of Kras*^G12V^*, GFP and Luc/mKate expression as well as homozygous deletion of p53 in the brain (Fig. 3A). Orthotopic intracranial Cre injection at 40 days of age (Marques et al., 2021) resulted in brain tumors in living mice carrying the Kras*^G12V^* allele detectable by longitudinal bioluminescence imaging (Fig. S3), while no fluorescence signal could be detected in this setting. Between 8 and 18 weeks post-Cre injection, mice that carried all four alleles displayed a detectable luminescence signal, unlike littermates that were wild-type for Kras (Fig. 3B, Fig. S3). Histological analyses of quadruple mutants at necropsy revealed well-demarcated forebrain tumors that expressed GFP, were largely negative against glial fibrillary acidic protein (GFAP) and positive for oligodendrocyte transcription factor 2 (OLIG2) (Fig. 3C,D).

Collectively, these data indicate that the *Col1a1*^LSL-effLuc-Kate^ reporter is suitable for detecting brain neoplasms *in vivo* and, to a lesser extent, prostate neoplasms *ex vivo*. The allele could be particularly useful for convenient and cost-efficient longitudinal brain tumor monitoring and for assessing treatment responses in preclinical trials instead of, or together with, MRI. Moreover, individual tumor cells could be isolated from tumor-bearing tissues by virtue of the fluorescence signal.

### Applicability in mouse models for pancreatic cancer

The potential usefulness of the *Col1a1*^LSL-effLuc-Kate^ reporter was next explored in the pancreas by using two tumor models. The reporter allele was first evaluated in the Rip1-Tag2 model for pancreatic neuroendocrine tumors (PanNET) (Hanahan, 1985; Saghafinia et al., 2021). To this end, the *Col1a1*^LSL-effLuc-Kate^ allele was combined with the RIP1-Tag2 and RIP7-Cre alleles, in which the rat insulin promoter drives expression of large T antigen and Cre recombinase, respectively, in pancreatic beta cells. At 8 weeks, a time point where pancreatic islets are dysplastic, no signal was observed in mice carrying all three alleles (Hanahan, 1985; Saghafinia et al., 2021). However, bioluminescence was apparent in the abdominal region of triple-allelic mice at 15 weeks, when macroscopic tumors are visible, and this was confirmed by *ex vivo* imaging of isolated pancreas (Fig. S4A). mKate fluorescence was not detected by using the IVIS in live mice, and the high red auto-fluorescence of the pancreas probably precluded *ex vivo* detection in skinned mice or isolated organs.

**Fig. 4.**
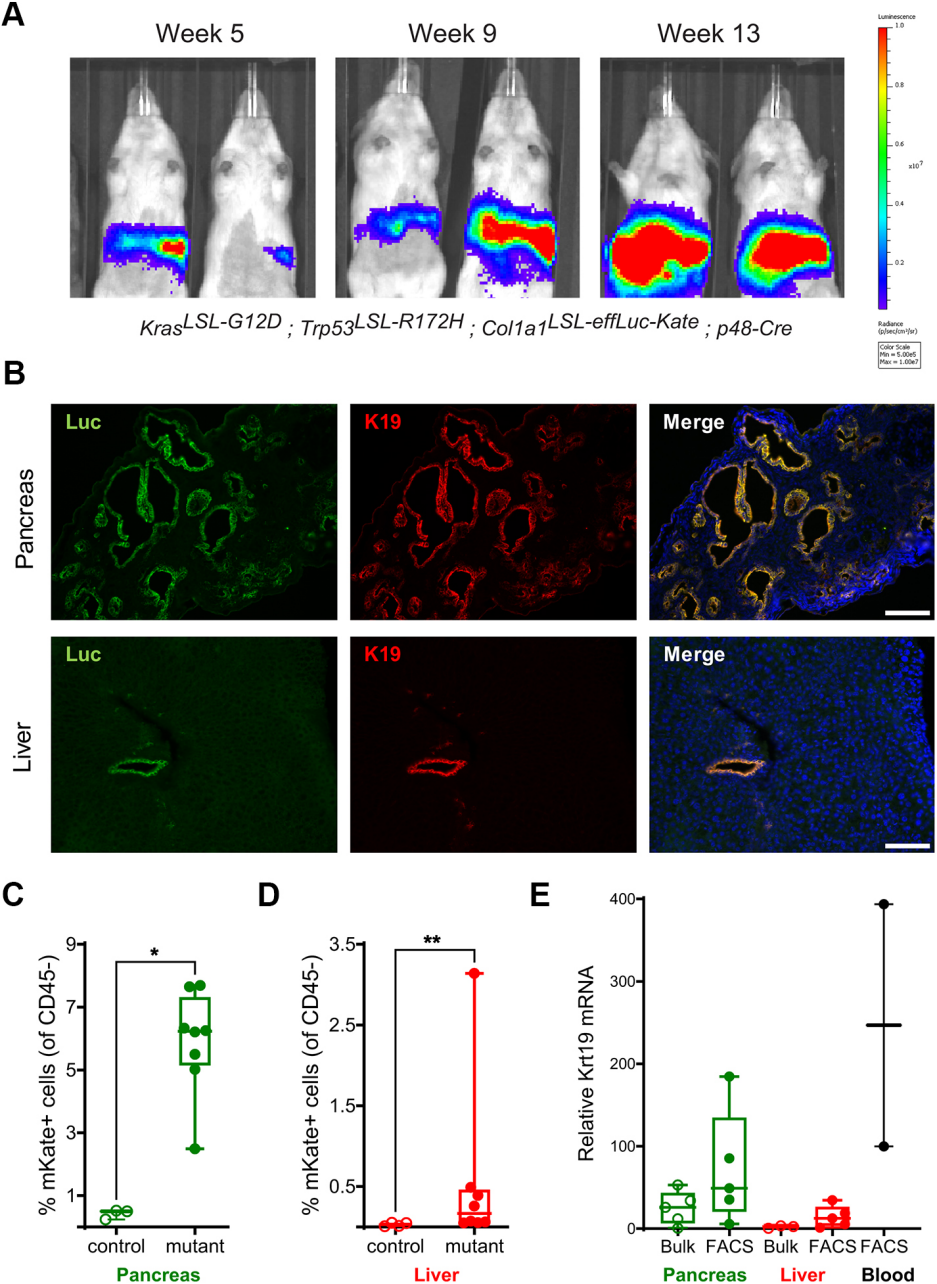
**Assessment of the LSL-effLuc-Kate dual reporter in a genetically engineered mouse model of pancreas cancer.** (A) Representative images of bioluminescence detection in male Kras^LSL-G12D^; Trp53^LSL-R172H^; p48-Cre^+^ mice carrying the *Col1a1*^LSL-effLuc-Kate^ dual reporter. Images were taken at age 5, 9 and 13 weeks (late PDAC stage) as indicated. For each time point, the same two mice are shown. (B) Immunofluorescence images of primary pancreatic tumors (top) and liver sections with micrometastases (bottom) stained for luciferase (Luc, green) and keratin 19 (K19, red). Images were taken using a representative Kras^LSL-G12D^; Trp53^LSL-R172H^; *Col1a1*^LSL-effLuc-Kate^; p48-Cre^+^ PDAC female mouse aged 15 weeks. Nuclei are counterstained with DAPI (blue). Scale bars: 100 µm. (C,D) Flow cytometric quantification of CD45-negative, mKate-positive cells in pancreas (C) and liver (D) of Kras^LSL-G12D^; Trp53^LSL-R172H^; *Col1a1*^LSL-effLuc-Kate^; p48-Cre^+^ mice at end stage (mutant: 13-15 weeks, male and female, *n*=8). Sex- and age-matched values from wild-type mice are shown for comparison (control, *n*=3). (E) Quantitative qRT-PCR analysis of *Krt19* mRNA expression in mKate-positive cells FACS-sorted from the pancreas, liver or blood of Kras^LSL-G12D^; Trp53^LSL-R172H^; *Col1a1*^LSL-effLuc-Kate^; p48-Cre^+^ mice at end stage (13-15 weeks), compared to bulk (pre-sorting) single cell suspensions of pancreas and liver. Data in C−E are shown as box-and-whisker plots around the median, *P<0.05, **P<0.01 (two-tailed unpaired Mann–Whitney test).

The dual reporter was next evaluated for luminescence-based longitudinal studies, and mKate-based metastatic cell detection and isolation in a model of pancreatic ductal cancer (PDAC). The *Col1a1*^LSL-effLuc-Kate^ allele was combined with the Kras*^LSL-G12D^*, Trp53*^LSL-R172H^* and p48-Cre alleles to allow simultaneous Kras*^G12V^* and Trp53*^R172H^* expression in progenitor cells of the mouse pancreas, and the subsequent development of PDAC (Hingorani et al., 2005; Ying et al., 2025). Bioluminescence of variable intensities was detected in the abdominal region of mutant mice as early as 5 weeks of age (Fig. 4A). *Ex vivo* imaging of isolated organs confirmed that the bioluminescence signal was originating from the pancreas (Fig. S4B). These data indicate that the *Col1a1*^LSL-effLuc-Kate^ reporter is suitable for bioluminescence detection of different pancreatic neoplasms *in vivo* and *ex vivo*.

In the PDAC model, constitutive p48-Cre expression induced expression of *Col1a1*^LSL-effLuc-Kate^ in normal pancreas and at early stages of pancreatic cancer development, i.e. of pancreatic intraepithelial neoplasia (PanIN), as revealed by an increase of bioluminescence imaging over time in live mice, consistent with tumor progression (Fig. 4A, Fig. S4C). However, the pattern was very heterogeneous and, in the mice with the highest increase, bioluminescence imaging decreased at later PDAC stages, when tumors are more desmoplastic (Fig. S4C). This result indicates that, although the *Col1a1*^LSL-effLuc-Kate^ reporter is functional in this PDAC model, it might not be suitable to monitor advanced/desmoplastic PDACs, at least not by using classic bioluminescence imaging technology. Rather, the reporter might be best in order to monitor early stages of tumor progression that, importantly, are not amenable to detection by the widely used method of ultrasound imaging (Beatty et al., 2011; Olson et al., 2011).

Histological analyses of methanol-fixed sections revealed keratin 19 (hereafter referred to as K19)/luciferase double-positive tumors in the pancreas as well as K19/luciferase double-positive liver micro-metastases (Fig. 4B). Moreover, mKate fluorescence was detectable by flow cytometry in the pancreas of all analyzed mutant mice, albeit in variable proportions. On average, 6% of the CD45-negative, non-hematopoietic cells and 50% of the epithelial cellular adhesion molecule (EpCAM)-positive epithelial cells had detectable mKate fluorescence (Fig. 4C, Fig. S4D,E). mKate fluorescence was also detected in liver cell suspensions, albeit only in 50% of the examined mice and in lower proportions (Fig. 4D). These data indicate that detecting the mKate signal in fresh tissue sections is possible by using a suitable high-resolution/sensitivity fluorescence microscope. A trend for increased mKate fluorescence was observed in blood (Fig. S4F), indicating the possible presence of circulating tumor cells (CTCs). This was confirmed by qRT-PCR analyses of CD45-negative/mKate-positive cells isolated by flow cytometry from pancreas, liver and blood, compared to the bulk (pre-sorting) population. Measurable gene expression of *Krt19* was found in mKate-positive cells isolated from blood, as well as liver and pancreas (Fig. 4E), together with *mKate* mRNA (Fig. S4G). These results warrant future investigation into the use of this reporter for detecting CTCs in blood, micro-metastases in the liver and their longitudinal correlation during neoplastic development. In sum, the *Col1a1*^LSL-effLuc-Kate^ reporter has the potential to complement the widely used ultrasound imaging technology that can detect macroscopic PDAC but not PanIN lesions or micro-metastases. Moreover, this reporter potentially aids the isolation of cancer cells from these lesions as well as from the blood – by using fluorescence-activated cell sorting (FACS) and magnetic beads – for multi-‘omics’ analyses.

### Conclusion

Understanding complex biological systems will ultimately contribute to the development of more effective therapies and improve patient outcomes. While GEMMs that recapitulate the molecular, cellular and systemic features of disease progression are instrumental for advancing our understanding of human diseases, such as cancer, it is important to include in such models the capability for disease detection and monitoring, as well as for purifying diseased cells. Despite some limitations, the reporter allele described here has features that will be of value to the biomedical research community. We have illustrated its applicability in GEMMs of bone, prostate, brain and pancreas cancer, as well as its limitations. Its utility can likely be further enhanced with more sensitive − for example, 3-dimensional − detection systems that better interrogate deep internal organs, such as the pancreas. Although demonstrated for cancer models, we envisage broader applicability for this reporter in studying development and organogenesis, homeostasis and aging, as well as other diseases in mice.

## MATERIALS AND METHODS

### Mice and treatments

All animal experiments were conducted according to institutional, national and European guidelines for animals used in biomedical research, and were approved by the Spanish, Austrian and Swiss Ethics Committees for Research and Animal Welfare.

The effLuc/mKate cassette encodes a fusion protein of enhanced firefly luciferase (effLuc) (Rabinovich et al., 2008) and the mKateS158A red fluorescent protein comprising a Ser158 to Ala point mutation, separated by a T2a ribosomal skipping site (Deniger et al., 2014). This cassette was cloned and inserted downstream a CAGGS−LSL cassette in the pBS31'RBGpA vector (Beard et al., 2006), removing the Tet-operator (TetO) sequence. The resulting construct was electroporated into KH2 (C57BL/6 x 129/Sv)F1 embryonic stem (ES) cells by the CNIO Mouse Genome Editing Core Unit according to (Beard et al., 2006), using a Flp-mediated single-copy-targeting procedure similar to the Tet-switchable Col1a1^TetO-Fos^ allele (MGI:5555845) used in this study (Briso et al., 2013; Bakiri et al., 2017). One correctly targeted ES cell clone that carried the *Col1a1*^LSL-effLuc-Kate^ allele was used to generate chimeras to transmit the *Col1a1*^LSL-effLuc-Kate^ allele. The Osx-Cre, Tyr^c-Brd^ (albino mutation), Rosa^LSL-rtTA-GFP^, Kras*^LSL-G12V^*, Trp53*^lox^*, Pten*^lox^*, Junb*^lox^*, Kras*^LSL-G12D^*, Trp53*^LSL-R172H^*, Psa-Cre, RIP1-Tag2, RIP7-Cre and p48-Cre alleles have been previously described (MGI:3689350, MGI:3640303, MGI:3583817, MGI:3582830, MGI:1931011, MGI:2156086, MGI:3036209, MGI:2136169, MGI:3039263, MGI:3761848, MGI:2429941, MGI:3603660, MGI:2387812). Mice were backcrossed and maintained on pure C57BL/6J, C57BL/6^c-^ (albino) or FVB backgrounds; unless otherwise indicated, littermates were used as controls. Mice were housed in a specific pathogen-free environment with free access to food and drinking water. Doxycycline (Dox; Sigma-Aldrich; 1 g/l) was supplied in sucrose-containing (100 g/l) drinking water. Orthotopic adenovirus delivery was performed as follow: Anesthetized 8-week-old mice were injected under the knee capsule with 10 µl of 10^8^ plaque-forming units (pfu) adeno-Cre virus (Ad5CMVCre, The University of Iowa Viral Vector Core). Intracranial or prostate delivery of Adeno-Cre was performed as described (Riedel et al., 2018; Thomsen et al., 2015; Marques et al., 2021) by using male mice aged 40 days or 7-week-old, respectively. All experiments−except those concerning brain and prostate models and which used males only − were carried out using male and female mice, and we did not notice any sex-related differences.

### *In vivo* and *ex vivo* imaging

Mice were anesthetized with a continuous flow of 1% to 3% isoflurane in 100% oxygen at a rate of 1.5 l/min. Isofluorane-anesthetized animals were imaged for luciferase and red fluorescence by using the IVIS 100/Xenogen (Fig. 4) or IVIS 200/Xenogen (Figs 1-3) systems 5-10 min after intraperitoneal injection of 2 mg of D-luciferin according to the manufacturer's specifications and by adjusting the settings to the minimum−maximum values of the most-intense sample. Imaged were the whole body or isolated organs from sacrificed mice using the same equipment within 1 h after live imaging − except for pancreas studies, for which organs were harvested, perfused with PBS and immersed in 5 mg/ml D-luciferin for 5 min before imaging. IVIS Living Image 3.2 Analysis software (PerkinElmer, UK) was used for data analysis . *Ex vivo* micro-computed tomography (micro-CT) was performed on non-decalcified formalin-fixed hindlegs using an eXplore Locus micro-CT scanner (GE Healthcare) and a Voxel 3D model built using MicroView (GE Healthcare).

### Histology

Tissues were frozen in O.C.T. compound (Tissue-Tek) or fixed in 4% formalin and embedded in paraffin before 4-8-µm sections were prepared. Bones containing knee tumors were decalcified in 10% EDTA for 3 weeks prior to embedding. Standard procedures were used for H&E staining. Immunohistochemistry (IHC) was performed either by the CNIO Histopathology Core Unit or manually as previously described (Thomsen et al., 2015; Marques et al., 2021; Bakiri et al., 2017; Hasenfuss et al., 2014) using matching secondary antibodies from the Vectastain Elite ABC kit (Vector Laboratories) and Carazzi's hematoxylin solution (Panreac Química S.L.U., Spain) for counterstaining. Antibodies used were against tRFP (Evrogen, detecting mKate, cat. no.: AB233; dilution 1:500), luciferase (Novus Biologicals, cat. no.: NB600-307; dilution 1:100), phosphorylated Akt (Cell Signaling Technology, cat. no.: 4060; dilution 1:200), GFP (Cell Signaling Technology, cat. no.: 2956; dilution 1:100), OLIG2 (Millipore, cat. no.: AB9610; dilution 1:500) and GFAP (DAKO, cat. no.: Z0334; dilution 1:1000). Pictures were taken with a Leica light microscope or using digital scans and Panoramic Viewer (3DHistech) or ImageJ software. For immunofluorescence, 10-mm-thick methanol-fixed sections were stained with antibodies against luciferase (Novus Biologicals cat. no.: NB100-1677, dilution 1:200) or keratin 19 (K19) (Troma III, Developmental Studies Hybridoma Bank, University of Iowa, cat. no.: AB 2133570; dilution: 1:1000), and counterstained with suitable fluorescence labeled secondary antibodies or DAPI (Roche, cat. no.: 10236276001). Sections were imaged using a Leica DM5500 microscope, an Olympus VS120 slide scanner, and a Zeiss Axioscan Z1 slide scanner. Images were processed with ImageJ, QuPath and the respective scanner-associated software.

### Cell culture and *in vitro* experiments

Murine primary ear fibroblasts were isolated using collagenase digestion of minced ethanol-sterilized ears collected from one 6-week old female chimera offspring heterozygous for the *Col1a1*^LSL-effLuc-Kate^ allele and cultivated a few days in DMEM supplemented with 10% fetal bovine serum at 37°C and 5% CO_2_. Next, cells were collected and plated in four independent 30-mm wells and adeno-Cre virus (Iowa University; 10^6^ pfu) was added to two of the wells. 48 h later, far-red mKate fluorescence was analyzed using a confocal microscope. Cells were subsequently lysed to measure luciferase activity using the Dual-Glo Luciferase Assay (Promega) and a Szabo Scandic luminometer following the manufacturer's recommendations, and 1/10 of the total lysates was used for the luciferase assay.

### Tissue processing for flow cytometry and molecular biology

Tumors and livers were harvested, minced with a scalpel and digested for 30 min with collagenase A (0.33 U/ml, Roche, cat. no.: 10103578001), Dispase II (0.85 U/ml, Roche, cat. no.: 4942078001) and DNAse I (144 U/ml, Roche, cat. no.: 4716728001) in RPMI medium (Gibco, cat. no.: 61870044) under intermittent shaking at 37°C. The resulting single-cell suspensions were passed through a 70-mm cell strainer. Peripheral blood was collected into EDTA-coated tubes and red blood cells were lyzed with PD Pharm Lyse buffer (BD Biosciences, cat. no.: 555899) according to the manufacturer's instructions. Cells were further used for flow cytometry, and cell sorting for qRT-PCR.

### Flow cytometry

Single-cell suspensions of livers, tumors or peripheral blood were incubated with anti-mouse CD16/32 antibody (BioLegend, cat no.: 101302; dilution 1:200) on ice for 15 min. Staining for antibodies against surface antigens was performed for 20 min on ice. Live/dead staining was performed for the isolation of live cells, using DAPI (Roche, cat. no.: 10236276001) or for tumor cell phenotyping, using fixable viability stain kit (Invitrogen, cat no.: L34964, dilution 1:1000) for 15 min on ice. The following antibodies were used: Ep-Cam-FITC (clone G8.8, BioLegend, cat no.: 118207, dilution 1:200), Ep-Cam-PerCP/Cyanine5.5 (clone G8.8, BioLegend, cat no.: 118219, dilution 1:200) and CD45-APC (clone 30-F11; BD Biosciences, cat no.: 559864, dilution 1:200). Samples were analyzed on a BD LSRFortessa (BD Biosciences) or a Gallios (Beckman Coulter) flow cytometer, and sorted using a FACS Aria III cell sorter (BD Biosciences). Data were processed using the FlowJo v10.8.0 software (BD Biosciences) and GraphPad Prism v9 and v10 (Graphpad/Dotmatics). The gating strategy is exemplified in Fig. S4D with the subsequent steps shown left to right.

### RNA isolation, reverse transcription and quantitative RT-PCR

RNA from sorted cells and tissues was isolated with the RNeasy Plus Micro Kit (Qiagen). A total of 150–250 ng RNA was used for cDNA synthesis using the PrimeScript RT Master Mix (RR036A, TaKaRa). qRT-PCR was performed using the Rotor-Gene SYBR Green Master Mix (Qiagen). Primers used were: *mKate* (forward: 5′-GACCCTTGGATGGGAAGCAT-3′; reverse: 5′-GTGACCTCCACCGACAAGTT-3′), *Krt19* (forward: 5′-ACCCTCCCGAGATTACAACC-3′; reverse: 5′-GGCGAGCATTGTCAATCTGT-3′), *Gapdh* (housekeeping, forward: 5′-AGGTCGGTGTGAACGGATTTG-3′; reverse: 5′-TGTAGACCATGTAGTTGAGGTCA-3′) and *Rpl13a* (housekeeping, forward: 5′-CTGTGAAGGCATCAACATTTCTG-3′; reverse: 5′-GACCACCATCCGCTTTTTCTT-3′). All procedures were performed according to the manufacturer's instructions.

### Statistics

Unless otherwise specified, data in plots and bar graphs are presented as the mean±s.e.m. or as box & whisker plots around the median. Statistical significance was determined with GraphPad Prism (version 9.4.1) using two-tailed unpaired Mann–Whitney test to compare two groups. Values of *P*<0.05 were considered statistically significant. Statistical significance is indicated as *P<0.05 and **P<0.01; ns indicates non-significance.

## Supplementary Material

10.1242/dmm.052130_sup1 Supplementary information
