# Supplementary material for: A new effLuc/Kate dual reporter allele for tumor imaging in mice
Source: Dis Model Mech. 2025 Jan 31;18(1):DMM052130. doi: 10.1242/dmm.052130 (PMC11789939; doi:10.1242/dmm.052130)
Supplement: Supplementary information [file dmm-18-052130-s1.pdf]

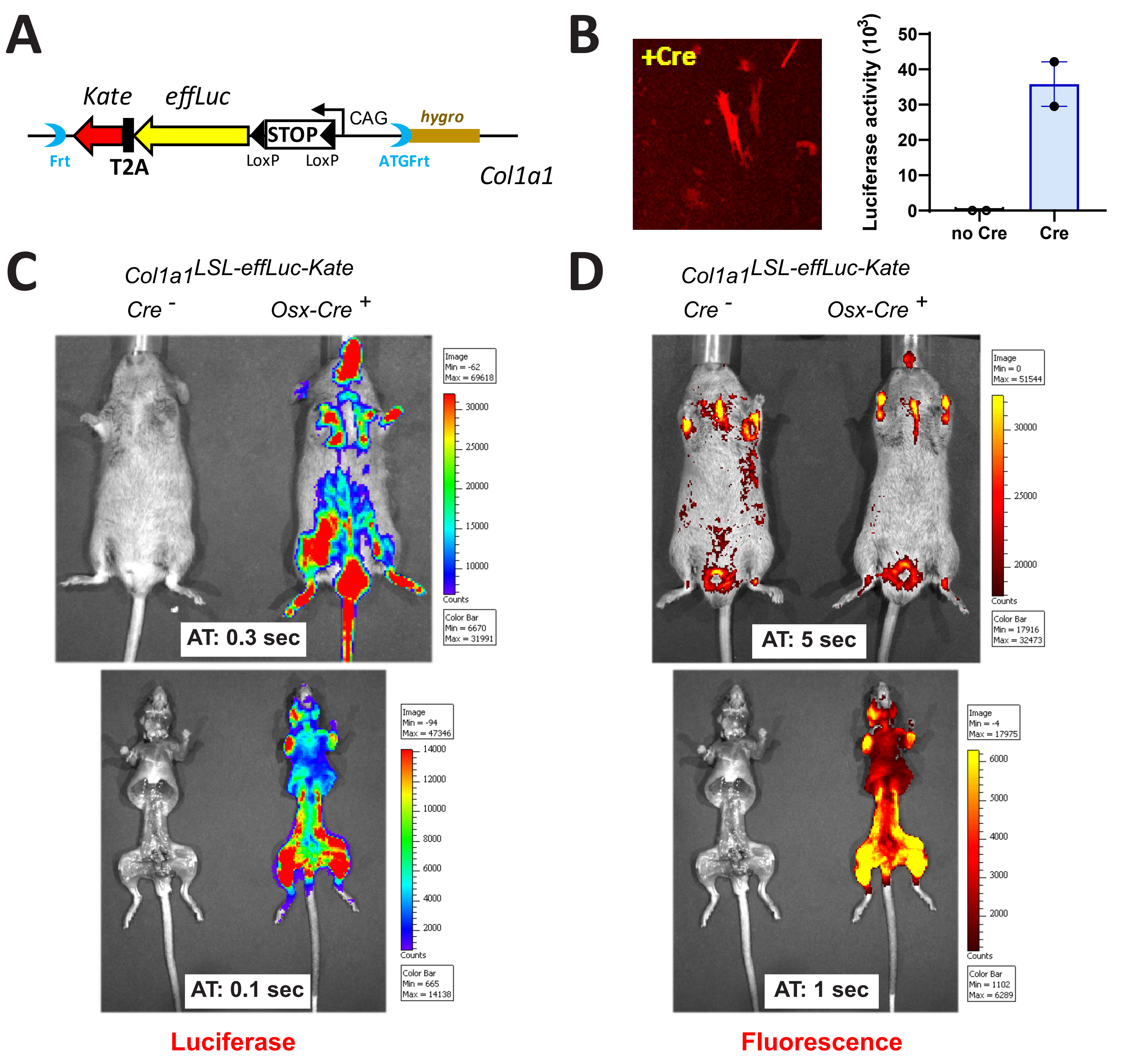

Fig. S1. Related to Fig. 1

A. Scheme of the *Col1a1* targeted allele: Flp-mediated recombination at the FRT ''homing'' site pre-targeted downstream of the ColA1 locus in KH2 ES cells resulted in the insertion of an ATG conferring hygromycin resistance and a CAG promoter driving the expression of enhanced firefly luciferase (effLuc) and mKate frt-hygro-pA, upon Cre/LoxP-mediated deletion of a floxed STOP sequence. CAG: cytomegalovirus (CMV) enhancer fused to the chicken beta-actin promoter; effLuc: enhanced firefly Luciferase; T2A: T2A ribosomal slip site; mKate S158A: enhanced mKate red fluorescence protein. **B.** Immunofluorescence and luciferase activity in primary adult ear fibroblasts isolated from a female chimera's offspring carrying the *Col1a1*$^{LSL\text{-}effLuc\text{-}Kate}$ allele 48 hours after in vitro Adeno-Cre infection. Left: Confocal image showing mKate fluorescence. Right: Luciferase activity in cell lysates compared to mock-infected cells. Bar = mean ± SD (n=2). **C-D.** Luminescence **(C)** and far red fluorescence **(D)** in live (top panels) mice and dissected skeletons (bottom panels). 18-week old male littermates carrying the *Col1a1*$^{LSL\text{-}effLuc\text{-}Kate}$ allele with or without Osx-Cre were imaged on an IVIS device (n=1 per genotype, experiment done twice). AT: signal acquisition time.

Disease Models & Mechanisms • Supplementary information

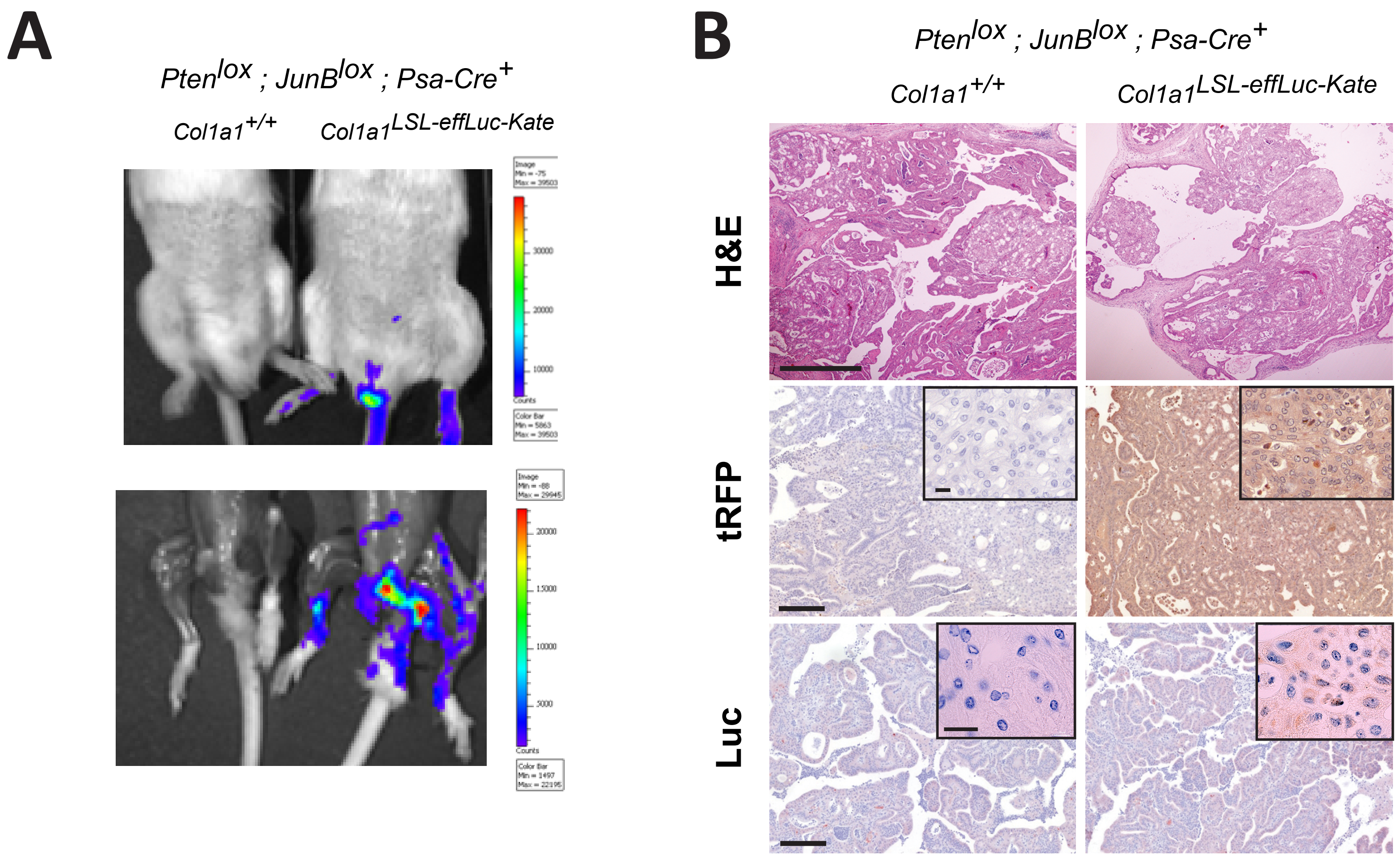

Fig. S2. Related to Fig. 2

A. Representative luminescence in live (top) mice and euthanized and skinned (bottom) mice. 43-week old male *Pten*^{lox}*; Junb*^{lox}*; Psa-Cre*$^{+}$ littermates carrying (right, n=2) or not (left, n=1) the *Col1a1*$^{LSL\text{-}effLuc\text{-}Kate}$ reporter allele were imaged on an IVIS device. **B.** H&E, TurboRFP (tRFP), detecting mKate and luciferase (Luc) IHC in prostate sections from the mice imaged in A. Bars: main panels: 200μm, inserts: 20μm.

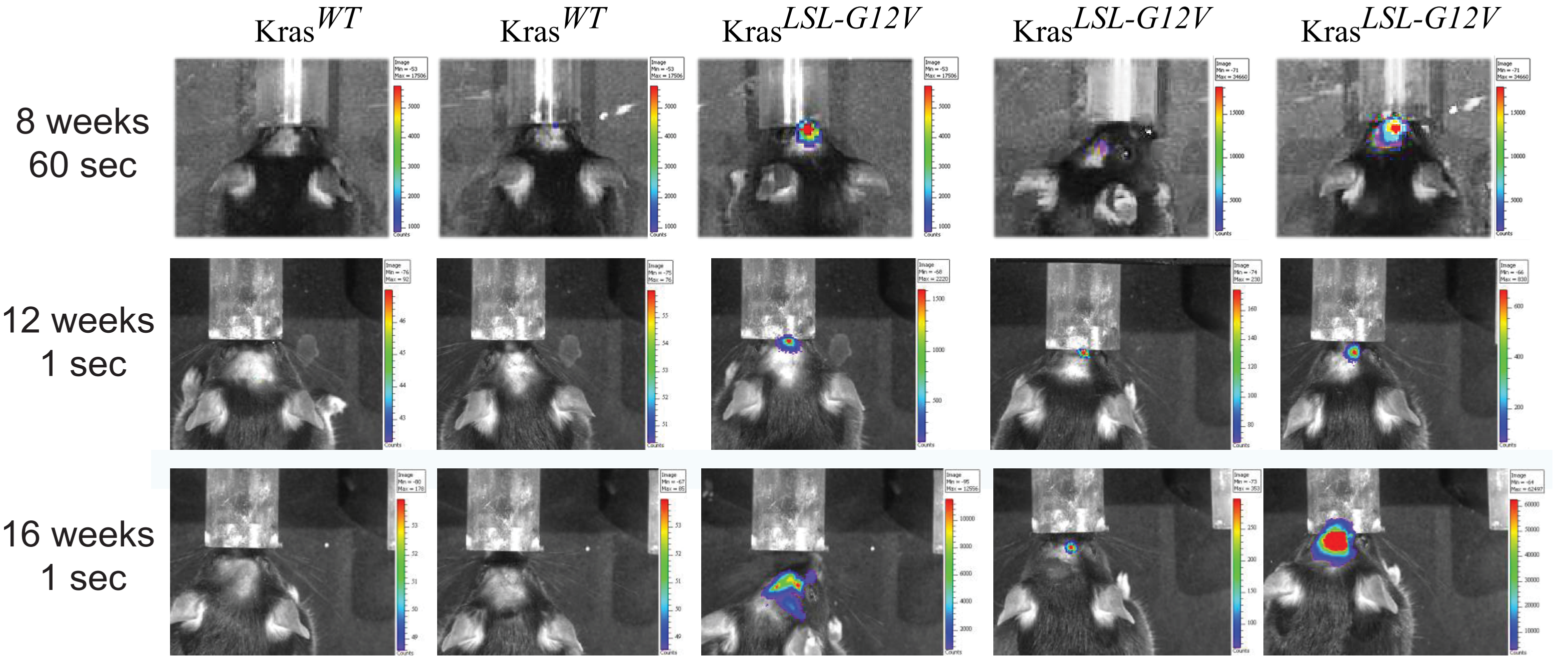

Fig. S3. Related to Fig. 3

Longitudinal IVIS imaging in 2 controls (Kras^{WT}) and 3 mutant (Kras$^{LSL\text{-}G12V}$) male mice homozygote for Trp53^{lox} and carrying the Col1a1$^{LSL\text{-}effLuc\text{-}Kate}$ and Rosa$^{LSL\text{-}rtTA\text{-}GFP}$ reporter alleles, imaged at different time points after orthotopic intracranial Adeno-Cre inoculation (2x10^8 pfu) at 40 days of age. Signal acquisition time is indicated. A total of n= 6 Kras^{WT} and n= 6 Kras$^{LSL\text{-}G12V}$ males from 3 independent cohorts and experiments were longitudinally followed.

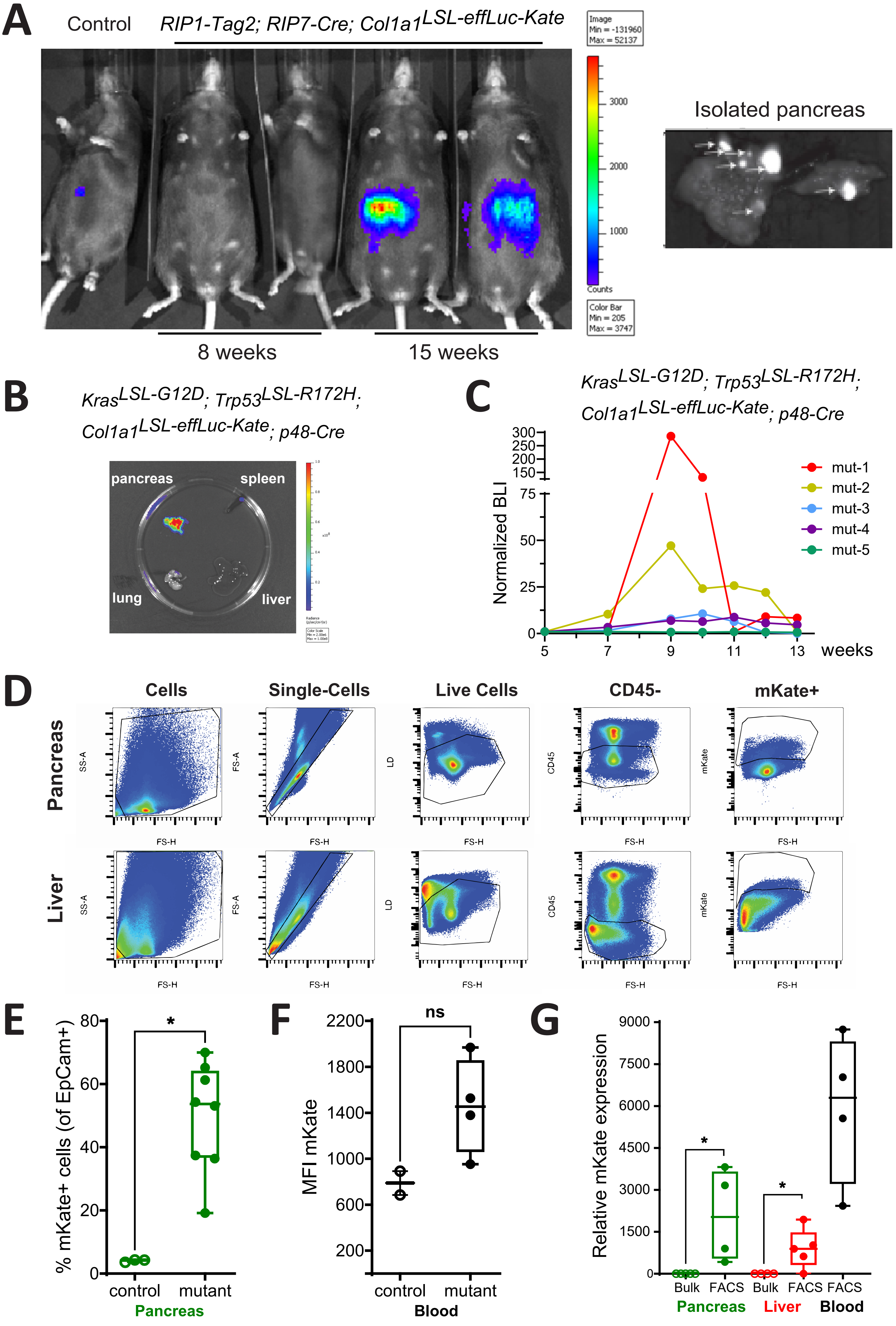

Disease Models & Mechanisms • Supplementary information

Fig. S4. Related to Fig. 4

A. Representative live BLI imaging of RIP1-Tag2; RIP7-Cre; *Col1a1*$^{LSL\text{-}effLuc\text{-}Kate}$ mice at the indicated time points (age in weeks, males and females). A 14 week-old wild-type mouse is included as a control for background luminescence. Insert: *Ex vivo* bioluminescence imaging of a pancreas isolated from a 15 week-old RIP1-Tag2; RIP7-Cre; *Col1a1*$^{LSL\text{-}effLuc\text{-}Kate}$ mouse. Arrows point to luminescence-emitting insulinomas. **B.** *Ex vivo* BLI imaging of pancreas, lung, liver, and spleen isolated from a representative; Kras$^{LSL\text{-}G12D}$; Trp53$^{LSL\text{-}R172H}$; p48-Cre-positive female mouse, carrying the *Col1a1*$^{LSL\text{-}effLuc\text{-}Kate}$ dual reporter, at 15 weeks (late-stage PDAC). **C.** Normalized BLI quantification of representative Kras$^{LSL\text{-}G12D}$; Trp53$^{LSL\text{-}R172H}$; *Col1a1*$^{LSL\text{-}effLuc\text{-}Kate}$; p48-Cre^{+} mice over time. n=5, each individual mouse is depicted in a different colour. **D.** Gating strategy (sequential from left to right) used in flow cytometry analyses and sorting in pancreas and liver samples. **E.** Flow cytometric quantification of EpCam/mKate double-positive cells in the pancreas of Kras$^{LSL\text{-}G12D}$; Trp53$^{LSL\text{-}R172H}$; *Col1a1*$^{LSL\text{-}effLuc\text{-}Kate}$; p48-Cre^{+} mice at end stage (mutant: 13-15 weeks, n=8, males and females). Sex- and age-matched wild-type mice are included for comparison (control, n=3). **F.** mKate Mean fluorescence intensity (MFI) in the blood of Kras$^{LSL\text{-}G12D}$; Trp53$^{LSL\text{-}R172H}$; *Col1a1*$^{LSL\text{-}effLuc\text{-}Kate}$; p48-Cre^{+} mice at end stage (mutant: 13-15 weeks, n=4, males and females). Sex- and age-matched wild-type mice are included for comparison (control, n=2). **G.** qRT-PCR quantification of mKate mRNA expression in mKate-positive cells FACS-sorted from the pancreas, liver and blood of Kras$^{LSL\text{-}G12D}$; Trp53$^{LSL\text{-}R172H}$; *Col1a1*$^{LSL\text{-}effLuc\text{-}Kate}$; p48-Cre^{+} mice at end stage (13-15 weeks). Expression in bulk (pre-sorting) single cell suspensions of pancreas and liver was undetectable and set to zero for plotting. n = 4-5. **F-G**: Data are plotted in box & whisker plots around the median, * $p < 0.05$, ns = not significant by two-tailed unpaired Mann Whitney test.

Disease Models & Mechanisms • Supplementary information
